# *MSS2* maintains mitochondrial function and is required for chitosan resistance, invasive growth, biofilm formation and virulence in *Candida albicans*

**DOI:** 10.1080/21505594.2020.1870082

**Published:** 2021-01-11

**Authors:** Cai-Ling Ke, Yu-Ting Liao, Ching-Hsuan Lin

**Affiliations:** Department of Biochemical Science and Technology, College of Life Science, National Taiwan University, Taipei, Taiwan

**Keywords:** *Candida albicans*, *MSS2*, mitochondria, chitosan, biofilms, virulence

## Abstract

*Candida albicans* is the most prevalent fungal pathogen in humans, particularly in immunocompromised patients. In this study, by screening a *C. albicans* mutant library, we first identified that the *MSS2* gene, an ortholog of *Saccharomyces cerevisiae MSS2* required for mitochondrial respiration, mediates chitosan resistance. Upon treatment with 0.2% chitosan, the growth of *mss2Δ* strains was strikingly impaired, and *MSS2* expression was significantly repressed by chitosan. Furthermore, *mss2Δ* strains exhibited slow growth on medium supplemented with glycerol as the sole carbon source. Similar to the chitosan-treated wild-type strain, the *mss2Δ* strain exhibited a significantly impaired ATP production ability. These data suggest that an antifungal mechanism of chitosan against *C. albicans* acts by inhibiting *MSS2* gene expression, leading to repression of mitochondrial function. Normal respiratory function is suggested to be required for fungal virulence. Interestingly, the *mss2Δ* mutant strains exhibited significantly impaired invasive ability *in vitro* and *ex vivo* but retained normal hyphal development ability in liquid medium. Furthermore, the *MSS2* deletion strains could not form robust biofilms and exhibited significantly reduced virulence. Collectively, these results demonstrated that the antifungal effect of chitosan against *C. albicans* is mediated via inhibition of mitochondrial biogenesis. These data may provide another strategy for antifungal drug development via inhibition of fungal mitochondria.

## Introduction

Mitochondria are specialized organelles found in eukaryotic cells. A primary role of mitochondria is to supply energy through the respiratory chain [1–3]. Moreover, mitochondria perform diverse functions, including roles in controlling the cell cycle, cell growth, signaling, ion homeostasis and metabolite production [2,4]. In humans, mitochondrial dysfunction might be associated with several human diseases [5–7]. In pathogenic fungi, inhibition of the respiratory chain influences the yeast-to-hyphal morphological transition, cell wall biogenesis, drug resistance and virulence [1,3,8–13]. However, the mechanistic link between mitochondria and the physiological roles of fungal pathogens remains largely unclear.

*Candida albicans* can reside in the human body as a commensal organism [14], but it is also the predominant fungal pathogen that can cause life-threatening systemic infections, particularly in immunocompromised patients [15,16]. The ability of *C. albicans* to colonize different niches and become pathogenic has been shown to be closely linked to its phenotypic plasticity [17–20]. In fact, several diverse types of *C. albicans* cells, such as yeast cells, hyphal cells, white cells, opaque cells, gray cells and gastrointestinally induced transition (GUT) cells, [19,21–24], also exhibit the striking ability of *Candida* cells for morphological transformation in response to different environments and host niches.

Among these phenotypic changes, the reversible morphological transition between yeast and hyphal forms is the central and defining virulence factor in *C. albicans* [21,25]. The yeast-to-hyphal transition is controlled by Cph1 and Efg1, the downstream targets of Cek1 MAP kinase and cyclic AMP (cAMP) signaling, respectively [26–31]. These signaling cascades and many downstream hypha-specific genes can be induced by different environmental conditions and components, such as high temperature, serum, N-acetylglucosamine (GlcNAc), CO_2_, and neutral pH [27,30,32–35]. Furthermore, the switch from the yeast to the hyphal form is highly associated with biofilm formation in hosts and on implantable medical devices [36,37], leading to resistance to clinical treatment.

Although mitochondria play major roles in the electron transport chain (ETC), energy production and cellular metabolism [1–3,10,13], information regarding the function of *C. albicans* mitochondria is currently very limited. In contrast to *Saccharomyces cerevisiae*, which lacks complex I (NADH:ubiquinone oxidoreductase), *C. albicans* and most other *Candida* species have five intact ETC complexes [38–40]. The contribution of ETC complex I to physiological roles is one of the most well studied topics in *C. albicans*. The NADH:ubiquinone oxidoreductases Nuo1 and Nuo2 are subunits of complex I and are conserved in several fungal pathogens [41]. *NUO1* or *NUO2* have been shown to be involved in complex I activity, cell wall biogenesis, hyphal formation and biofilm development [9,41]. Additionally, *GOA1*, originally identified through mutant library screening to be required for oxidant adaptation and present in all members of the CTG clade of pathogenic *Candida* species except *C. glabrata*, is involved in mitochondrial complex I activity [42]. Furthermore, the *GOA1* deletion mutant showed lower respiratory activity, a reduced ability to form hyphae, high sensitivity to antifungal drugs and cell wall alterations and, moreover, was required for full virulence [41–44]. Further studies showed that the three transcription factors (TFs) Rbf1, Hfl1 and Dpb4, which positively regulate *GOA1* directly or indirectly, are involved in mitochondrial biogenesis [12]. Moreover, a recent study identified that several potential mitochondrial proteins involved in the expression of ETC complex I, III or IV are required for respiration, antifungal susceptibility and virulence [13]. However, the functions of most subunits of each ETC complex and mitochondrial proteins have not been studied in C. *albicans*.

Chitosan, a deacetylated unit of chitin, has numerous commercial applications and biomedical uses [45–50]. Additionally, the positive charge of chitosan endows it with considerable antimicrobial activity against bacteria and fungi if the pH is below 6.5 [51–55]. Although we have identified many transcription factors that potentially mediate chitosan resistance in *C. albicans* via screening of a TF mutant library [56], the mechanisms underlying the antifungal action of chitosan against *C. albicans* remain largely unknown. Here, we further screened a library of protein kinase mutant strains of *C. albicans* to understand the potential cascades required for the response to chitosan. *MSS2* was identified from this screen. The *MSS2* gene was first identified in *S. cerevisiae* and is required for ETC activity and mediates membrane insertion of the C-terminus of Cox2p, a subunit of cytochrome c oxidase (complex IV) [57–59]. However, the function of *MSS2* in *C. albicans* is unknown. In this work, we demonstrated that *C. albicans MSS2* is necessary for chitosan tolerance and that its expression is inhibited by chitosan. Furthermore, *mss2Δ* cells exhibited a significantly decreased respiration ability compared with wild-type (WT) cells, similar to that seen in the chitosan-treated wild-type strain. These data suggested that an antifungal mechanism of chitosan against *C. albicans* acts by inhibiting mitochondrial biogenesis. Interestingly, *mss2Δ* strains showed significantly impaired invasive growth, biofilm formation and virulence. Hence, inhibition of respiratory activity could be a promising therapeutic strategy to control *C. albicans* infection.

## Materials and Methods

### Media and reagents

The media used in these experiments included yeast extract-peptone-dextrose (YPD) medium, YPD medium containing 200 μg/ml of nourseothricin (Werner BioAgents, Jena, Germany), and Spider medium, and they were prepared as previously described [60]. In YPG medium, dextrose was replaced with 3% glycerol. The base RPMI 1640 liquid medium, RPMI 1640 medium supplemented with 0.2% acetic acid (final pH 6.3, buffer control medium), and chitosan medium prepared from RPMI 1640 supplemented with 0.2% chitosan dissolved in 0.2% acetic acid (experimental medium) were prepared as previously described [56,61,62]. The chitosan used in this study was purchased from Shin Era Technology (Taipei, Taiwan). The molecular weight (MW) of chitosan is approximately 20–35 kDa, and its degree of deacetylation is approximately 90% [56]. All chemicals were purchased from Sigma-Aldrich Chemical unless stated otherwise.

### Mutant library screening

The *C. albicans* mutant library strains derived from BWP17 were purchased from different laboratories [63–67]. Each *C. albicans* kinase mutant strain was grown separately in YPD liquid medium in 96-well microplates at 30°C overnight. The strains were then transferred to 96-well microplates containing RPMI 1640 liquid medium (supplemented with 0.165 M MOPS and 2% glucose), buffer control medium (RPMI 1640 medium supplemented with 0.2% acetic acid) or experimental medium (RPMI 1640 supplemented with 0.2% chitosan dissolved in 0.2% acetic acid). The 96-well microplates were incubated at 30°C for 48 hr, and the optical density of the wells was then measured at 600 nm in a SpectraMax 190 microplate reader (Molecular Devices, CA, USA). The absorbance values of each mutant treated with chitosan or acetic acid were compared to determine the susceptibility of the strains to chitosan, and statistical significance was determined by Student’s t-test.

### Plasmid and strain construction

The *C. albicans* strains and oligonucleotides used in this study are listed in Tables S1 and S2, respectively.

To construct the *mss2Δ* strains, the 5ʹ flanking and 3ʹ flanking regions of *MSS2* were PCR-amplified using primer pairs 1139/1140 and 1141/1142, respectively. The 5ʹ and 3ʹ PCR products were digested with *Apa*I/*Xho*I and *Sac*II/*Sac*I, respectively, and cloned into the plasmid pSFS2A [68] to generate the plasmid pSFS2A-*MSS2* KO. The plasmid was linearized with *Apa*I/*Sac*I and transformed into the wild-type strain SC5314 to generate the heterozygous *mss2Δ*/*MSS2* mutant strains. The *SAT1* marker was recycled by treatment with 2% maltose. The heterozygous strains were retransformed with the same deletion construct to generate the *mss2Δ/mss2Δ* strains YL1700/YL1701.

The *MSS2* complementation construct was generated by amplification of its endogenous promoter and open reading frame (ORF) using the primer pair 1710/1711. The PCR product was digested with *Sac*II/*Sac*I and cloned into pSFS2A to generate pSFS-*MSS2* AB. The plasmid was partially digested with *Bsm*I and transformed into *mss2Δ* to generate YL1986/YL1987.

### Sensitivity assays

Ten-fold serial dilutions of *C. albicans* cells were generated from suspensions with an OD_600_ of 1.0. A 5 µl volume of each dilution was spotted onto RPMI 1640 agar, RPMI 1640 agar containing 0.2% acetic acid, or RPMI 1640 agar supplemented with 0.2% chitosan. Images were acquired after the plates were incubated at 30°C for 2–3 days. To test the cell membrane- and cell wall-perturbing agents, final concentrations of 0.04% SDS, 60 μM calcofluor white and 1.6 μg/ml caspofungin were used. Images were acquired after the plates were incubated at 30°C for 2–3 days.

### Determination of mitochondrial activities via Seahorse XFe 24 assays

A Seahorse XF analyzer (XFe 24) was used to measure the oxygen consumption rate (OCR) following a previous protocol with slight modifications [69,70]. Overnight cultures of *C. albicans* cells (4 × 10^5^ cells/well in 50 μl) were washed twice with PBS and distributed into poly-D-lysine-precoated (50 μg/ml) 24-well XFe microplates (Seahorse Bioscience). Four replicates of each strain and chitosan treatment condition were established per experiment. Samples were incubated for 1 hr at 30°C to allow for cell adhesion. The untreated groups were incubated with 50 μl of RPMI 1640 medium, and the treated groups were incubated with 50 μl of 0.2% chitosan for 20 min. Each well was filled with 575 μl of RPMI 1640 medium before analysis. The energy pathway (indicated by the OCR) of each sample was measured using the Seahorse XFe 24 at 9 min intervals. Mitochondrial respiration was first induced by assay medium containing 2 mM L-glutamine. Then, 100 mM triethyltin bromide (TET), 5 μM FCCP and 2 mM antimycin A were sequentially injected into the assay wells during the analyses. Student’s t-test was used for the statistical analyses.

### Quantitative reverse transcription-PCR

The assay was performed in accordance with the established protocol in our laboratory [71]. Overnight cultures of *C. albicans* cells (200 μl) were transferred into 10 ml of fresh YPD. Cells were harvested by centrifugation and washed 3 times with sterile water. Cells of each sample were treated with RPMI 1640 medium, RPMI 1640 medium containing 0.2% acetic acid, or RPMI 1640 medium containing 0.2% chitosan at 30°C for 5, 10 or 20 min. Furthermore, to measure the expression of the six core regulatory genes required for biofilm development, cells were collected from biofilms in a silicone model as described in the “Biofilm assays” section of the Materials and Methods. Cells were harvested and washed three times with sterile water. Total RNA was isolated from each sample using a MasterPure^TM^ Yeast RNA Purification Kit (Epicenter, Madison, WI, USA), and DNA was removed with DNase I (Thermo Fisher Scientific, Waltham, MA, USA). RNA was transcribed to cDNA with an iScript^TM^ cDNA Synthesis Kit (Bio-Rad Laboratories., Inc., CA., USA). Quantitative PCR was performed in a Bio-Rad CFX Manager (Bio-Rad Laboratories, Hercules, CA, USA). Each experiment was independently repeated three times, and the means of the data from the triplicate experiments are shown. The differences between the control group and the experimental group were analyzed with Student’s t-test. The primer pairs 541/542, 1197/1198, 1781/1782, 1783/1784, 1785/1786, 1787/1788, 1791/1792, and 1789/1790 were used to detect *ACT1, MSS2, BCR1, BRG1, ROB1, EFG1, TEC1* and *NDT80* expression, respectively. Student’s t-test was used for the statistical analyses. The expression of the target genes was normalized to the expression of the *ACT1* gene.

### Hyphal formation assays

The wild-type, *mss2* mutant and complemented strains were grown in RPMI 1640 for 12 hr or in YPD supplemented with or without 50% serum for 4 hr. Hyphal development of the wild-type, *mss2* mutant and complemented cells was examined with an Eclipse Ti inverted microscope (Nikon Instruments Inc., Melville, NY, USA).

### Agar invasion assays

Overnight cultures of *C. albicans* cells at an OD_600_ of 1.0 were subjected to 10-fold serial dilution. A 2 µl volume of each dilution was spotted onto an RPMI 1640 agar plate. The plates were incubated for 4 days at 30°C. Colonies above the agar surface were washed with sterilized deionized water as previously described [72]. Images were acquired before and after the washes.

### Cell culture

HeLa (human epithelial cells from a fatal cervical adenocarcinoma) cells were cultured in DMEM (Dulbecco’s Modified Eagle Medium, Thermo Fisher) supplemented with 10% fetal bovine serum (FBS) and antibiotics (10 μg/mL streptomycin and 100 U/mL penicillin) and then incubated at 37°C with 5% CO_2_.

### Cell invasive assays

The experimental procedures of the cell invasive assays followed the established protocol with slight modifications [73,74]. Overnight cultures of HeLa cells on the glass coverslips were washed with PBS and incubated with 5 × 10^5^ *C. albicans* cells at 37°C with 5% CO_2_ for 1 hr. Samples were fixed with 4% paraformaldehyde for 30 min. Then, the samples were incubated with 25 μg/mL concanavalin A–fluorescein conjugate (Invitrogen) in PBS for 45 min to stain *C. albicans* cells localized outside the epithelial cells because concanavalin A-fluorescein cannot penetrate cell membrane of HeLa cells. The membranes of epithelial cells were subsequently permeabilized with 0.1% Triton X-100 detergent for 15 min. Calcofluor white (10 μg/mL, 0.1 M Tris-HCl) was then used to stain *C. albicans* cells localized outside of and inside epithelial cells for 20 min. Samples on coverslips were washed with PBS three times and then mounted with 100% glycerol. An Eclipse Ti inverted microscope was used to examine the fluorescence of each sample. Three experiments were performed, and 100 *C. albicans* cells were analyzed in each experiment. Then, the percentage of *C. albicans* cells that had invaded the epithelial cells was calculated.

### Biofilm assays

Biofilm assays were performed following the previously established protocol for measuring biofilm biomass in a silicone model [75]. Silicone squares (Bentec Medical, PR72034-06 N, 1.5 cm × 1.5 cm) placed in a 12-well plastic plate were sterilized and weighed before incubation in bovine serum (Sigma, B-9433). The plates were placed in a shaking incubator at 150 rpm and 37°C overnight. The serum-treated silicone squares were washed with 2 ml of PBS and placed in new 12-well plates supplemented with 2 ml of Spider medium. Notably, 1% mannitol in the Spider medium was replaced with 1% glucose for the *C. albicans* biofilm analyses because *mss2* mutant strains could not utilize mannitol and survive under these culture conditions. Approximately 2 × 10^7^ cells from the overnight YPD cultures of the *C. albicans* SC5314 wild-type, *mss2* mutant and complemented strains were placed on top of the silicone squares. The inoculated samples were incubated at 37°C with shaking at 150 rpm for 90 min to allow for adhesion. The samples were washed with 2 ml of PBS and then incubated in 2 ml of fresh Spider medium for 60 hr at 37°C with shaking at 150 rpm. The supernatant was removed from each sample, and the silicone squares were dried overnight to determine the dry weight of the biofilms.

### Virulence assays and fungal burden assessment

Five-week-old male ICR mice purchased from BioLASCO (Taiwan) were used (n = 9). Overnight cultures of *C. albicans* cells in YPD medium were harvested by centrifugation. Cells from each sample were washed three times with PBS. Mice were inoculated with *C. albicans* cells of the wild-type, *mss2Δ* and complemented strains (5 × 10^5^ cells in 200 µl) via tail vein injection. The course of infection was monitored for 25 days to assess survival. Statistical significance was set at a *P* value of < 0.05 using the log-rank test.

For the fungal burden assessment, mice were infected intravenously with wild-type or mutant *C. albicans* cells (5 × 10^5^ cells in 200 µl) and sacrificed three days post infection. Kidneys (n = 4 per group) and brains (n = 4 per group) harvested from the mice were dissected and weighed. Each organ was placed in 10 ml of PBS solution and homogenized for 2 min at 30,000 rpm with IKA dispersers (IKA, T10 basic ULTRA-TURRAX® Crushing Disperser, Germany). The tissue homogenates were serially diluted on YPD agar plates supplemented with 100 µg/ml chloramphenicol. The plates were incubated at 30°C for 24 to 48 hr to determine the CFUs per gram of kidney or brain tissue homogenate.

### Statistical analyses

For the virulence assays, statistical significance was set at a *P* value of < 0.05 using the log-rank test. Statistical analyses of data from other experiments were performed using a one-tailed or two-tailed Student’s t-test with a 95% confidence interval.

## Results

**Determination of the involvement of *MSS2* in chitosan resistance by screening a library of protein kinase mutant strains of *C. albicans***

**Figure 1.**
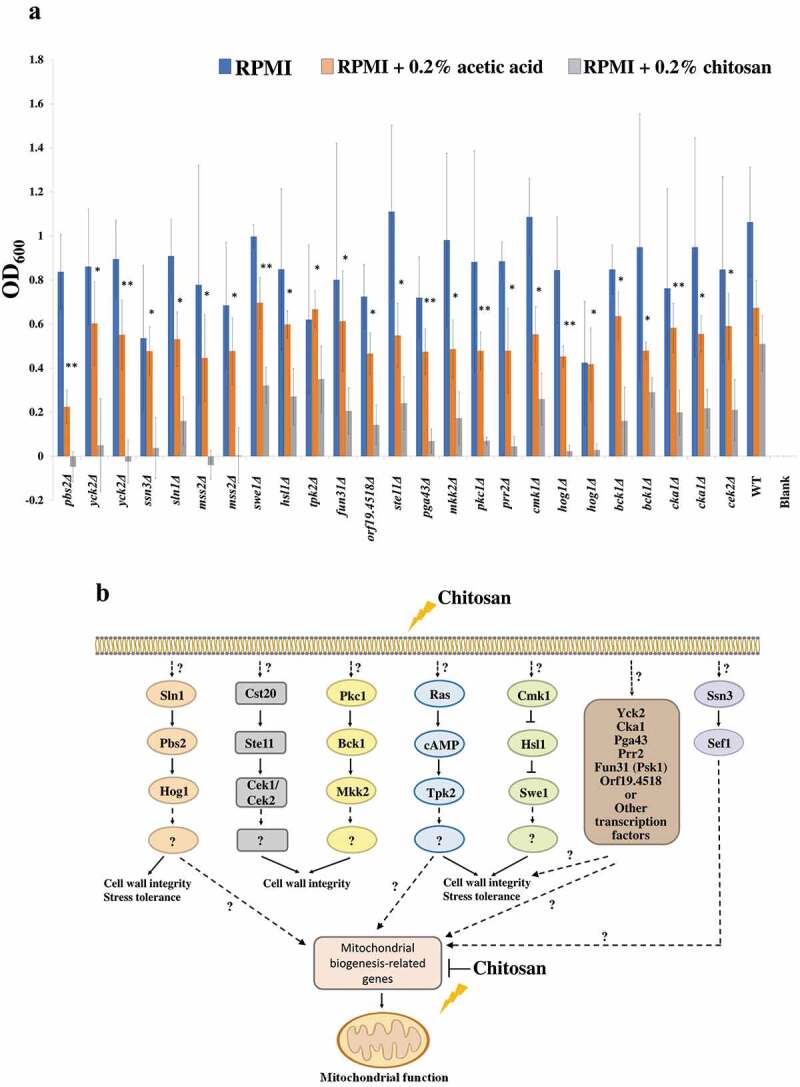
**Screening results of a library of protein kinase mutant strains of *C. albicans* during chitosan treatment**. (a) Each mutant strain was grown separately in 96-well microplates in RPMI 1640 liquid medium (RPMI), RPMI containing 0.2% acetic acid or RPMI containing both 0.2% acetic acid and chitosan. The optical density of the cells after incubation at 37°C for 48 hr was measured at 600 nm. Each mutant strain treated with acetic acid or chitosan was compared to determine the susceptibility of the strains to chitosan. Statistical significance was evaluated by Student’s t-test (unpaired, two-tailed)., P<0.05; P<0.01. (b) Diagrammatic illustration of the potential signaling cascades and factors involved in chitosan resistance in *C. albicans*. In addition to the electrostatic interaction between chitosan and the *C. albicans* cell surface, the intracellular response of several potential pathways and factors could be associated with chitosan. These cascades, such as Hog1, Cek1/Cek2, Mkc1, Ras1-cAMP, and calcineruin pathways and other factors [56], are associated with the cell wall or cell membrane biogenesis, stress tolerance, and mitochondrial function, which are required for chitosan resistance. The functional annotation of each gene is listed in the Table S3 (worksheet 2)

A total of 166 kinase mutant strains were tested with or without chitosan treatment (Supplementary Table S3 worksheet 1). Twenty-one kinase gene mutants exhibited a significant growth defect upon chitosan treatment (Figure 1a and Supplementary Table S3 worksheet 1). Screening data showed that several important signaling pathways are involved in the chitosan response. The potential cascades and factors involved in chitosan resistance are summarized in Figure 1b [56]. These pathways included the Hog1 (*sln1Δ, pbs2Δ* and *hog1Δ*), Cek1/Cek2 (*ste11Δ* and *cek2Δ*), Mkc1 (*pkc1Δ, bck1Δ* and *mkk2Δ*) MAP kinases, Ras1-cAMP (*tpk2Δ*) and calcineurin (*cmk1Δ, swe1Δ* and *hsl1Δ*) pathways, as the indicated mutants listed in parentheses were highly susceptible to chitosan (Figure 1 and Supplementary Table S3 worksheet 2). These findings are not surprising, because it has been proposed that chitosan binds to the fungal surface [52,55,56], and these signaling pathways have been shown to mediate cell wall and cell membrane integrity in *C. albicans* or budding yeast [76–80]. Additionally, *YCK2, FUN31* (*PSK1*) and *CKA1* have been shown to be important for cell surface biogenesis and drug resistance [66,81,82]. The function of three genes, namely, *ORF19.4518, PGA43* and *PRR2*, remains unclear (Figure 1 and Supplementary Table S3 worksheet 2). Interestingly, several lines of evidence in the screening data showed that the chitosan used in this study targets mitochondria. First, two independent *mss2Δ* strains exhibited significantly impaired cell growth upon chitosan treatment (Figure 1a and Supplementary Table S3), although the role of *MSS2* in *C. albicans* has not yet been determined. Second, the Ssn3-regulated gene *SEF1* is involved in mitochondrial iron metabolism [83]. Third, both the Ras1-cAMP and Hog1 MAP kinase pathways have been shown to be important for maintaining mitochondrial activity [84,85]. Based on these observations and the finding that its optical density was the lowest observed in the screen (Figure 1a and Supplementary S3), *MSS2* was selected for further investigation.

**The *MSS2* gene is involved in chitosan susceptibility, and its expression is repressed by chitosan in *C. albicans* SC5314**

**Figure 2.**
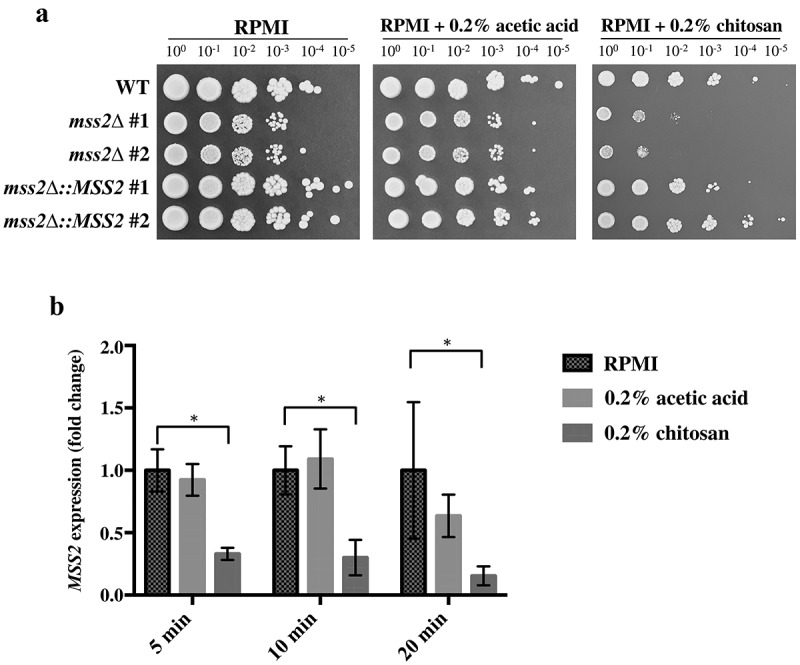
***C. albicans* MSS2 mediates chitosan resistance, and its expression is repressed by chitosan**. (a) The mss2 null mutant exhibited significant growth defects on medium containing 0.2% chitosan, and complementation restored the resistance to chitosan. A mild growth defect of mss2*Δ* was observed in the RPMI and buffer control (0.2% acetic acid) groups. (b) Treatment with 0.2% acetic acid for 5, 10 and 20 min did not significantly affect MSS2 expression, whereas SC5314 cells treated with chitosan for each duration exhibited significant reductions in MSS2 expression. The values are presented as the means ± SDs of at least three experimental replicates and were compared with the value for untreated wild-type SC5314 cells. Expression levels were normalized to those of the ACT1 gene. Statistical significance was determined using Student’s t-test, P < 0.05

To verify the previous results, *MSS2* was knocked out in the *C. albicans* SC5314 strain, and the chitosan susceptibility of the mutant strains was analyzed. As shown in Figure 2a, the *mss2Δ* strains in the RPMI 1640 and buffer control (RPMI 1640 + 0.2% acetic acid) groups exhibited a mild growth defect, whereas the *mss2Δ* strains exhibited more severe growth impairment on medium supplemented with 0.2% chitosan. Chitosan resistance was restored in the complemented strains. These results indicate that *C. albicans MSS2* is required for chitosan tolerance. Furthermore, quantitative polymerase chain reaction (qPCR) analysis showed that *MSS*2 expression was significantly repressed in the wild-type strain after chitosan treatment for 5, 10 and 20 min compared with that in untreated *C. albicans* cells (Figure 2b).

### *Respiratory activity is repressed in both* mss2Δ *cells and chitosan-treated wild-type cells*

**Figure 3.**
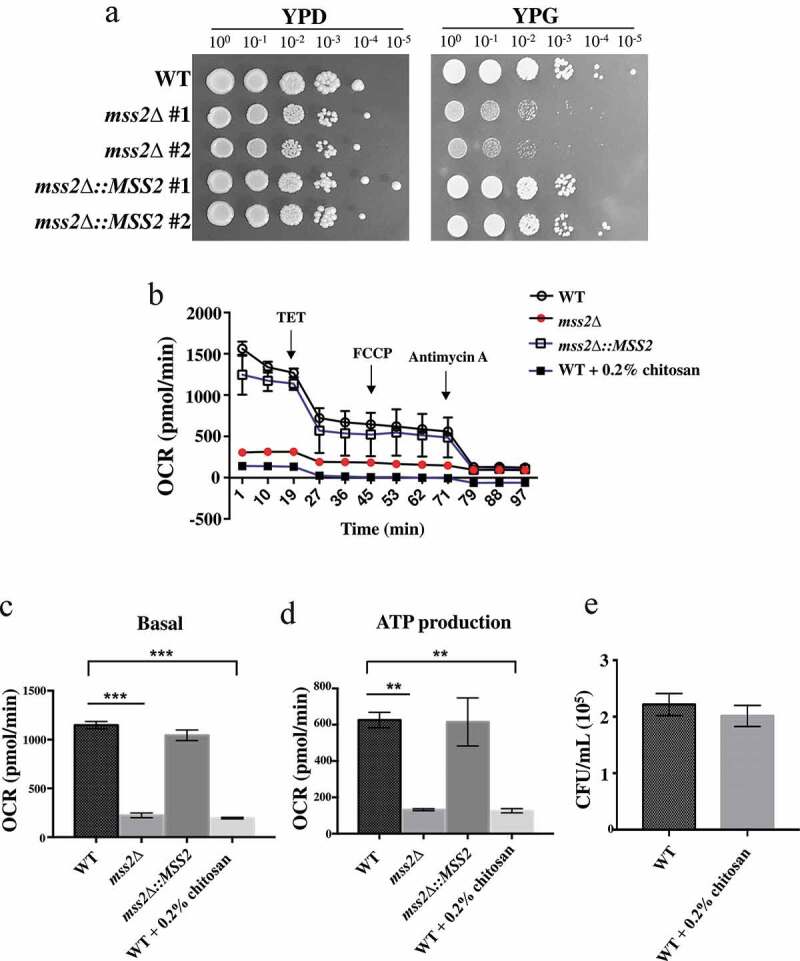
***MSS2* deletion mutants cannot efficiently utilize glycerol as the sole carbon source and exhibit strikingly impaired respiratory activity**. (a) Compared to the wild-type strain, the *mss2Δ* strains showed growth defects on YPG medium. (b) Mitochondrial respiratory activity in C. albicans cells was assessed by extracellular flux analysis. Mitochondrial respiration was assessed by measuring the OCR after sequential addition of 100 mM TET, 2 μM CCCP, 5 μM FCCP and 2 mM antimycin A. Quantification of respiratory activity under (c) basal and (d) ATP production conditions, with reference to the mitochondrial respiration data obtained in B. (e) The CFUs were similar between the untreated and chitosan-treated WT cells. The results are shown as the average of the values from three independent experiments and are the mean ± SD values. **, P < 0.01; ***, P < 0.001

The exact role of *MSS2* in *C. albicans* remains unknown. In the budding yeast *S. cerevisiae*, deletion of the Sc*MSS2* gene reduced mitochondrial biogenesis, as evidenced by the failure to integrate the C-terminus of Cox2p, a subunit of complex IV, in the mutant strain [57,59]. We therefore investigated whether the *C. albicans MSS2* gene is involved in the ETC. As shown in Figure 3a, *mss2*Δ strains exhibited more severe growth defects in yeast extract-peptone-glycerol (YPG) medium than in other media, suggesting that *C. albicans MSS2* is involved in mitochondrial biogenesis and energy production. To provide support for this hypothesis, a Seahorse analyzer was used to measure mitochondrial respiration. The *mss2*Δ strain exhibited a significantly decreased OCR under both basal respiration and ATP production conditions (Figure 3b, 3c and 3d). Mitochondrial function was restored in the complemented strain (Figure 3b, 3c and 3d). To determine whether chitosan treatment causes the death of wild-type and/or *mss2Δ* cells by causing severe mitochondrial deficiency, the viability of the wild-type cells treated with chitosan was assessed. Figure 3e shows that the CFUs of the wild-type and *mss2Δ* strains were similar after chitosan treatment for 30 min. These data suggest that an antifungal mechanism of chitosan against *C. albicans* acts by inhibiting mitochondrial biogenesis. Notably, addition of the uncoupler carbonylcyanide p-trifluoromethoxyphenylhydrazone (FCCP) was not sufficient to attain maximal respiration; thus, the maximal respiratory capacity and spare respiratory capacity could not be calculated (Figure 3b, 3c and 3d). This effect might have occurred because FCCP is used mainly to study mammalian cells. As described previously, chitosan repressed *MSS2* expression (Figure 2b). These findings imply that chitosan-treated wild-type cells might display mitochondrial activity similar to that seen in the *mss2Δ* strain. Indeed, compared to the other cells, chitosan-treated wild-type cells exhibited a significantly reduced OCR under both basal respiration and ATP production conditions (Figure 3b, 3c and 3d). These data suggest that chitosan exerts an antifungal effect against *C. albicans* by inhibiting its respiratory system.

**Deletion of *MSS2* in *C. albicans* increases susceptibility to sodium dodecyl sulfate (SDS) but confers resistance to calcofluor white**

**Figure 4.**
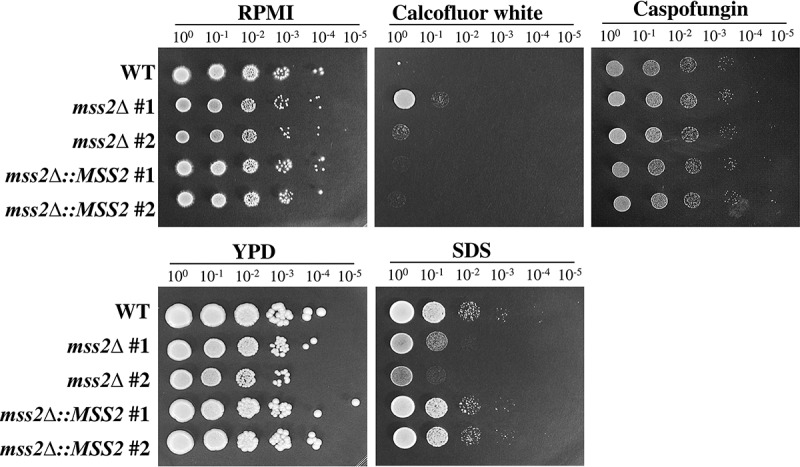
***MSS2* deletion enhances resistance to calcofluor white and sensitivity to SDS**. The *mss2Δ* strains did not exhibit changes in caspofungin sensitivity but showed increased resistance to the cell wall-perturbing agent calcofluor white and impaired growth on medium containing SDS

A functional link between the cell wall and mitochondria has been reported in several review articles [1–3,8,10,11]. We therefore sought to determine whether *C. albicans MSS2* is involved in cell wall integrity. Calcofluor white has been shown to interfere with cell wall assembly by targeting chitin, leading to an increase in cell wall stress [86,87]. The primary target of the antifungal drug caspofungin in *C. albicans* is β-1,3-glucan synthase [88]. In contrast, SDS is an amphipathic molecule that has both hydrophilic and hydrophobic regions and damages membranes and lipids [89]. Calcofluor white and caspofungin (cell wall-perturbing agents) and SDS (membrane-perturbing agent) were therefore selected as treatments in cell growth assays. As shown in Figure 4, the *mss2Δ* strains exhibited significant growth inhibition on medium supplemented with 0.04% SDS but showed better growth than the other strains on medium supplemented with 60 μM calcofluor white. In addition, the *mss2Δ* strains showed no difference in growth on medium supplemented with 1.6 μg/ml caspofungin compared to RPMI 1640 medium. These data suggest that *MSS2* might be involved in cell wall biogenesis. However, the growth of *mss2Δ* on medium supplemented with calcofluor white was different from that of the *goa1Δ* and *nuo2Δ* strains, which were highly sensitive to calcofluor white [12,41,42,44].

***mss2Δ* mutant strains exhibit a significantly impaired invasive growth ability on semisolid RPMI 1640 agar and in epithelial cells**

**Figure 5.**
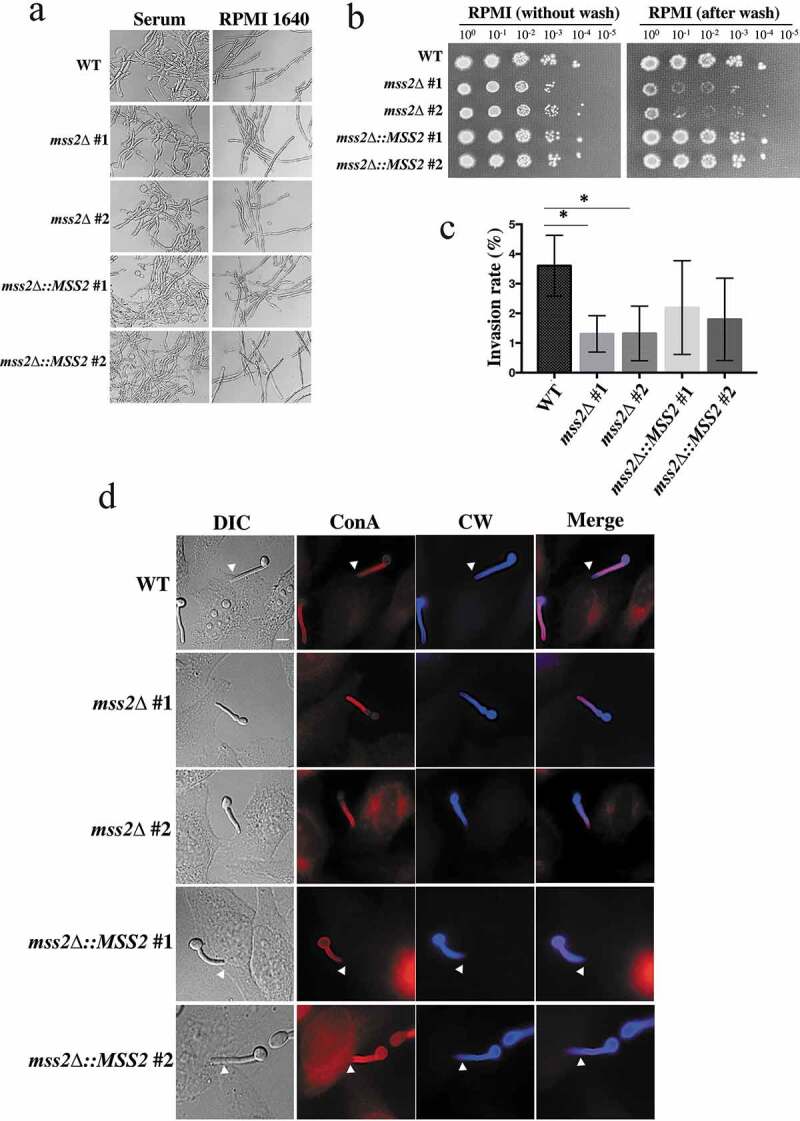
***Mss2δ* mutant strains exhibit a significantly impaired invasive growth ability on solid agar**. (a) *MSS2* is not involved in hyphal formation in YPD + serum and RPMI 1640 liquid media. (b) *MSS2* deletion strains of *C. albicans* show severe defects in invasive growth on semisolid RPMI 1640 agar. (c) Quantitative analysis of the invasion assays of HeLa cells shows that the presence of *MSS2* in *C. albicans* is able to reinforce the invasion of HeLa epithelial cells. Values are the mean ± SD from three replicates. *, *P* < 0.05. (d) Representative images of *C. albicans* hyphae-invaded HeLa cells. Extracellular hyphae were stained with Concanvalin A (red), whereas both extra- and intracellular hyphae were stained with calcofluor white (blue) after the cell membranes of HeLa cells were permeabilized with Triton X-100. The arrows show hyphae invasion. Scale bar: 10 μm

Mitochondrial dysfunction can impair hyphal development, as deletion of *GOA1, NUO1* and *NUO2* results in decreased hyphal formation [41,42]. We sought to determine whether the *mss2Δ* strains exhibit similar phenotypes. However, the *mss2Δ* strains exhibited no differences in hyphal formation in YPD + serum and RPMI 1640 liquid media (Figure 5a). Interestingly, that the *mss2Δ* strains exhibited less filamentous growth than the wild-type strain on RPMI 1640 semisolid agar. The results of agar invasion assays further demonstrated that the *mss2Δ* strains were more easily washed out than the wild-type and complemented strains (Figure 5b). We further evaluated whether *C. albicans MSS2* is involved in the invasion of HeLa epithelial cells. Concanavalin A and calcofluor white were used to differentiate hyphae that contacted the epithelial cell surface and hyphae that had actually invaded the epithelial cells. As shown in Figures 5c and 5d, hyphae of the wild-type strain exhibited stronger invasive ability than those of *mss2Δ* strains, and the difference was statistically significant. The *MSS2*-complemented strains had partially recovered invasion rates (Figure 5c), implying that invasion ability might be *MSS2* expression-dependent. These data indicate that *MSS2* is required for invasive growth on semisolid agar surfaces and in epithelial cells.

### MSS2 *deletion strains cannot form robust biofilms*

**Figure 6.**
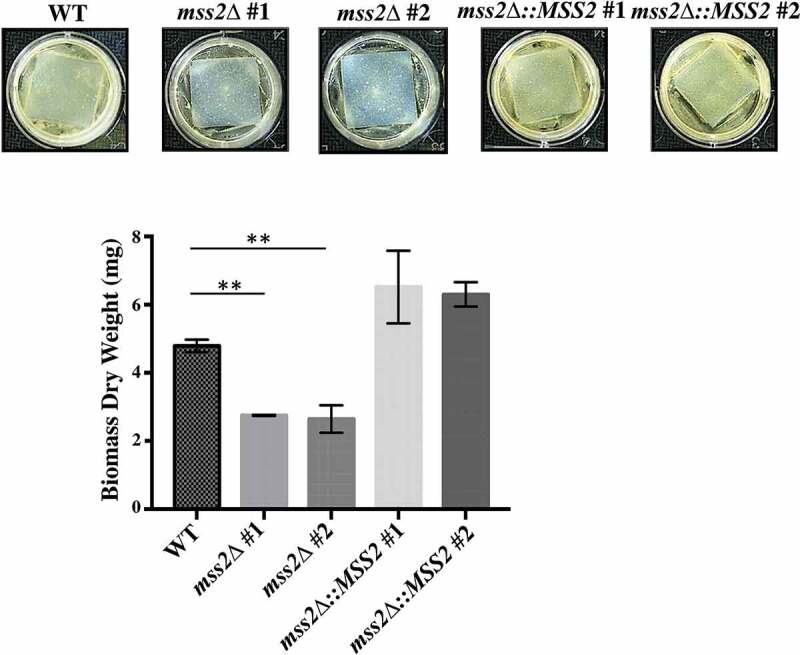
***MSS2* deletion strains cannot form robust biofilms**. The results of biofilm assays with the *C. albicans* wild-type, *mss2Δ* and complemented strains on silicone squares showed that the *MSS2* gene is necessary for biofilm development. The presented values are the means ± SDs of the values from three independent experiments with three replicates each. **, *P*< 0.01

Given that hyphal development on solid surfaces is a key event in successful biofilm formation by *C. albicans*, biofilm formation was evaluated in the *mss2Δ* strains. Compared with the wild-type strain (5.62 mg), the *mss2Δ* strains exhibited a significant reduction in biofilm biomass (2.99 and 2.56 mg) (Figure 6). Reintroduction of a functional *MSS2* gene into each *C. albicans mss2Δ* mutant restored biofilm formation (7.19 and 7.37 mg) (Figure 6).

### *Mss2 regulates the expression of the core biofilm regulatory genes* TEC1 *and* NDT80

**Figure 7.**
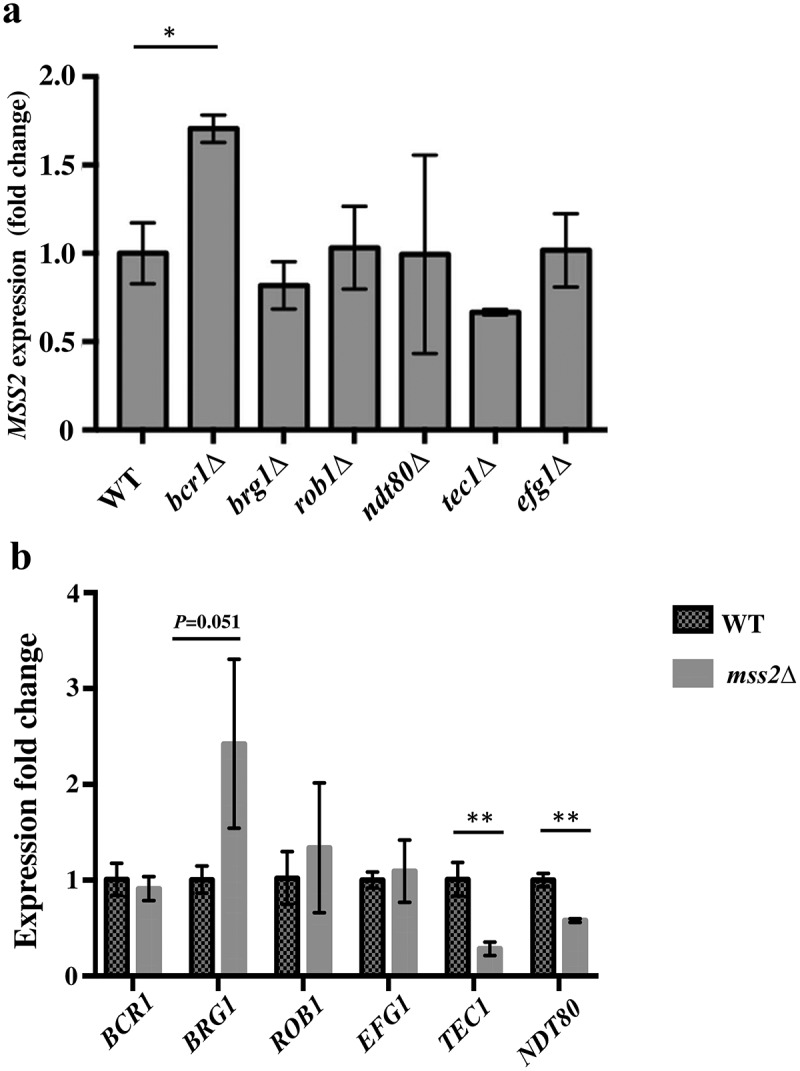
**Mss2 positively regulates *TEC1* and *NDT80* expression during biofilm formation**. (a) Loss of any one of the biofilm genes did not affect *MSS2* expression, whereas (b) the expression of *TEC1* and *NDT80* was significantly reduced in the *mss2Δ* strain. *, *P*< 0.05

Central to the understanding of the mechanisms underlying *C. albicans* biofilm formation is the understanding that biofilm growth is regulated by a complex regulatory network involving several major transcription factors [16,17,18]. We further investigated whether six core biofilm regulatory factors in *C. albicans* regulate *MSS2* expression and, conversely, whether Mss2 regulates the expression of these six factors. As shown in Figure 7a, deletion of *BRG1, ROB1, NDT80, TEC1* or *EFG1* did not significantly affect *MSS2* expression during the biofilm development stage, whereas *bcr1Δ* induced *MSS2* expression. Furthermore, *TEC1* and *NDT80* were significantly repressed in the *mss2* null mutant (Figure 7b). These data demonstrated the role of *MSS2* in biofilm formation.

### MSS2 *gene deletion significantly reduces fungal virulence*

**Figure 8.**
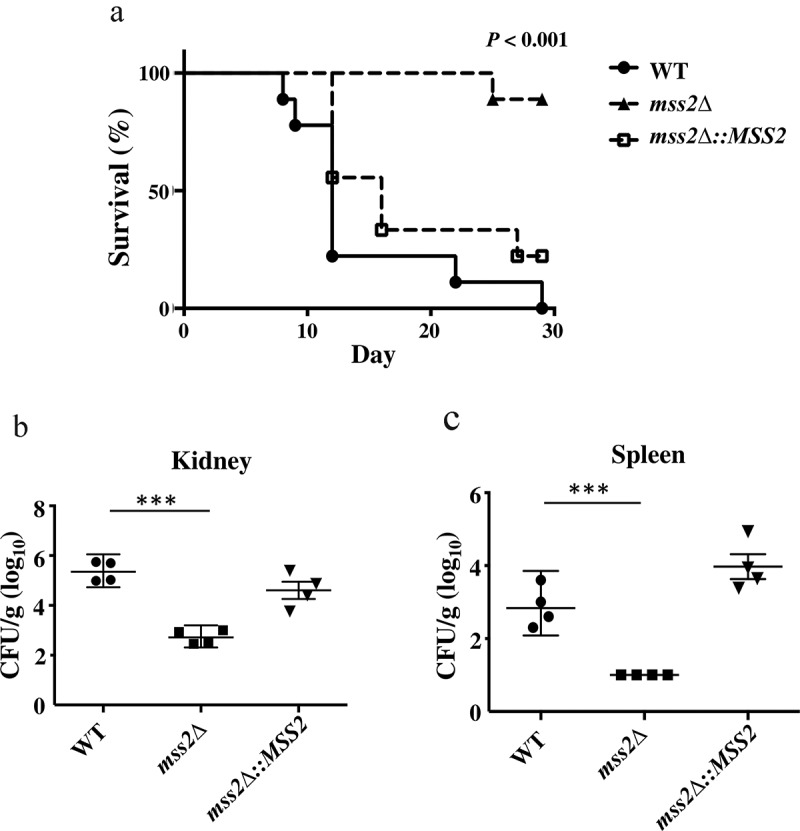
***MSS2* deletion strains exhibit reduced virulence and result in lower fungal burdens**. (a) Survival curves of mice after infection with 5 × 10^5^ *C. albicans* cells revealed that *mss2Δ* is involved in pathogenicity. The fungal burden in the kidney (b) and spleen (c) in four mice per strain was measured on day 3 post infection with C. albicans. *, *P*< 0.05; **, *P*< 0.01

The hyphal and biofilm formation abilities are the predominant virulence factors of *C. albicans* [90,91]. Thus, we further determined whether the *C. albicans mss2* null mutant was associated with attenuated virulence in a murine model. As shown in Figure 8a, the virulence of the *mss2Δ* strain was significantly decreased compared to that of the wild-type strain (*P* < 0.001, log-rank test). Reintroduction of a functional *MSS2* gene into the *mss2Δ* strain resulted in recovery of virulence. We further analyzed the fungal burden in murine kidneys and spleens. The fungal burdens were significantly decreased in the kidney (*P* < 0.01) and spleen (*P* < 0.05) in mice infected with the *mss2Δ* strain (Figures 8b and 8c).

## Discussion

Mitochondria function as a master organelle for energy production. The ability to utilize different carbon sources in glucose-limited environments and host niches is critical for the survival of fungal pathogens. However, mitochondria are also the central hub that coordinates multiple aspects of fungal biology, such as cell wall biogenesis, metabolite production, biofilm development, drug resistance and virulence [1–3,8,9,13,41,42,44,92]. However, the exact mechanisms underlying the ability of mitochondria to manipulate their flexibility to respond to changing environments remain largely unknown. *C. albicans* is a predominant pathogen affecting humans, particularly immunocompromised patients [15,16]. In this study, we identified 21 genes required for chitosan resistance by screening a library of protein kinase mutant strains. One antifungal mechanism of chitosan acts by targeting the microbial cell surface, leading to an inhibitory effect [52,55,56,93–95]. Indeed, 15 of the 21 genes were found to be involved in cell wall and cell membrane integrity (Supplementary Table 3), as the library mutants exhibited increased sensitivity to chitosan. Few studies have reported that chitosan might target mitochondria in *C. albicans* and *S. cerevisiae* [54,94]. Here, the *MSS2* gene, an ortholog of *S. cerevisiae MSS2*, was determined to be involved in chitosan resistance in *C. albicans*. The functional role of *MSS2* in *C. albicans* has not been studied. In *S. cerevisiae*, loss of *MSS2* causes improper assembly of Cox2 [57–59]. Thus, *MSS2* might also control the assembly of the *C. albicans* cytochrome IV subunit proteins (protein complex) and be required for respiratory activity. To the best of our knowledge, this report is the first to clarify the role of *MSS2* in *C. albicans* and provide direct evidence that the antifungal mechanism of chitosan acts against this fungus by antagonizing mitochondrial activity. However, we cannot rule out the combinatorial effects of several signaling pathways required for the chitosan response, which may thereby contribute to the impacts of chitosan.

Although the links between cell wall biogenesis and mitochondrial function remain largely unknown, mitochondrial dysfunction resulting in fungal cell wall defects has been reported [1,10,12,41,42,44]. Specifically, mutants with loss of *GOA1, NOU1, NOU2, NDH51, RBF1* or *HFL1* are sensitive to calcofluor white and other cell surface-perturbing agents [12,41,42,44]. Furthermore, *GOA1, NUO1, NUO2* and *NDH51* are involved in cell wall-related pathways [41,42,44]. Our results showed that the growth of the *mss2Δ* strains was not affected upon caspofungin treatment, suggesting that the of cell wall β-1,3 glucan content was unaffected in the mutant strains. Interestingly, unlike mutants of the known mitochondrial genes, *mss2* null mutants exhibited enhanced resistance to calcofluor white (a cell wall-perturbing agent) but sensitivity to SDS (a cell membrane-perturbing agent). These data suggest that the cell membrane of *mss2Δ* might be more vulnerable and that the cell wall of *mss2Δ* might contain more chitin, as calcofluor white binds mainly to chitin [86,87]. These results further indicate that the growth inhibitory effect of chitosan on the *mss2Δ* strains could be due to damage to the cell membrane and mitochondrial biogenesis.

The ability of *C. albicans* to undergo the yeast-to-hyphal transition is important in disseminated candidiasis and tissue invasion [21,25]. Unlike the well-known signaling pathways mediating this morphological transition, the precise mitochondrial functions involved in the complex cellular pathways that control hyphal development remain obscure. In general, mitochondrial dysfunction inhibits hyphal formation in *C. albicans* [1,3,41,42], but the effect of respiratory deficiency on filamentation varies with the inducing environment [9,42]. Interestingly, we found that *mss2Δ* retained a normal filamentation ability in both YPD + serum and RPMI 1640 liquid medium but exhibited a striking defect in invasive ability *in vitro* and *ex vivo*, which may lead to reduced biofilm formation and virulence and have a potential impact on the mucosal infection of this mutant. These data suggest that in addition to the yeast-to-hyphal transition, specific regulatory mechanisms are required for invasive growth on abiotic and biotic surfaces. Indeed, adhesion, turgor-driven force and proteolytic enzyme-mediated mechanisms are directly involved in successful invasion of host cells [96]. Although many genes have been shown to be required for invasive growth on contact surfaces, most genes are also associated with hyphal development in liquid medium; these genes include *RAS1, CDC24, RSR1, BUD2, HWP1, MKC1* and many others [97–99]. Interestingly, *C. albicans RHR2*, encoding a glycerol 3-phosphatase, is required for maintaining intracellular hydrostatic pressure (turgor pressure). *RHR2* deletion strains exhibit no defect in hyphal formation [100] but do exhibit adherence defects and reduced biofilm formation [101,102]. Similarly, *bcr1Δ* exhibits normal hyphal growth in liquid medium but reduced virulence due to its substrate adhesion deficiency and decreased biofilm formation [64,103]. The cell wall protein Dfip is involved in both invasive growth on semisolid agar and virulence, but the null mutant exhibits normal hyphal formation and adhesion ability [104]. These data suggest that *MSS2* deletion might repress invasive growth, thereby impacting on biofilm formation and virulence. How *MSS2* mediates the invasion process during interactions with abiotic and biotic surfaces and what genes are specifically involved in this process remain open questions.

Biofilms are highly associated with *Candida* infections. In *C. albicans*, a core complex regulatory network is critical for biofilm formation [75,105,106]. Here, we showed that the *mss2Δ* mutant exhibited significantly downregulated expression of *TEC1* and *NDT80*. Previous reports have shown that *TEC1* is involved in the interaction with epithelial cells [97], suggesting that the *MSS2* deletion strain might exhibit reduced adherence ability *in vivo*. In addition, we found high levels of *MSS2* expression in *bcr1Δ*. The results imply that the adherence-related transcription factor Bcr1 regulates *MSS2* directly or indirectly [88]. Furthermore, the *C. albicans ssn3Δ* strain displays enhanced biofilm formation and mitochondrial respiration compared with the wild-type strain [107]. Thus, the reduced biofilm formation of the *mss2Δ* strains may arise from mitochondrial deficiency. Notably, it has been suggested that mitochondrial activity is necessary and beneficial for biofilm formation in the early stage, as the results of some transcriptomic and metabolomic studies showed decreased mitochondrial activity in mature biofilms [75],^105^[108].

Mitochondria in *C. albicans* are important contributors to energy production, virulence, drug resistance and cell wall integrity; thus, the respiratory chain is an attractive target for the development of drugs against human fungal pathogens [1,2,10]. Currently, numerous respiratory chain inhibitors have shown considerable antifungal activity [3,11]. Chitosan is a biodegradable and nontoxic natural polysaccharide [50,93], and its broad-spectrum antifungal mechanisms that could target the cell surface and mitochondria and exert several intracellular actions against human pathogens endow it with substantial commercial potential [52,54–56,61,93]. These factors indicate that chitosan is a promising molecule for future antifungal drug development either alone or in combination with current antifungal drugs.

## Supplementary Material

Supplemental Material
