# Supplementary material for: MSS2 maintains mitochondrial function and is required for chitosan resistance, invasive growth, biofilm formation and virulence in Candida albicans
Source: Virulence. 2021 Jan 11;12(1):281–97. doi: 10.1080/21505594.2020.1870082 (PMC7808435; doi:10.1080/21505594.2020.1870082)
Supplement: Supplemental Material [file KVIR_A_1870082_SM4574.zip › supplement/tables3.docx]

Here is the Table S3 URL link. (<https://docs.google.com/spreadsheets/d/16zCVIw21zZLm70LFa4LMr7tkHhPBCmC-/edit#gid=571833204>)
