# Supplementary material for: MSS2 maintains mitochondrial function and is required for chitosan resistance, invasive growth, biofilm formation and virulence in Candida albicans
Source: Virulence. 2021 Jan 11;12(1):281–97. doi: 10.1080/21505594.2020.1870082 (PMC7808435; doi:10.1080/21505594.2020.1870082)
Supplement: Supplemental Material [file KVIR_A_1870082_SM4574.zip › supplement/Table_S1.docx]

**Table S1. Strains used in this study.**

| **Strain** | **Species** | **Genotype** | **Source** |
| --- | --- | --- | --- |
| YL2 | *C. albicans* | wild-type SC5314 strain | 1 |
| YL1700 | *C. albicans* | *mss2∆* | This study |
| YL1701 | *C. albicans* | *mss2∆* | This study |
| YL1986 | *C. albicans* | *mss2∆::MSS2* | This study |
| YL1987 | *C. albicans* | *mss2∆::MSS2* | This study |

1. **Bennett RJ, Johnson AD.** 2006. The role of nutrient regulation and the Gpa2 protein in the mating pheromone response of *C. albicans*. Mol Microbiol **62:** 100-119.
