# Supplementary material for: MSS2 maintains mitochondrial function and is required for chitosan resistance, invasive growth, biofilm formation and virulence in Candida albicans
Source: Virulence. 2021 Jan 11;12(1):281–97. doi: 10.1080/21505594.2020.1870082 (PMC7808435; doi:10.1080/21505594.2020.1870082)
Supplement: Supplemental Material [file KVIR_A_1870082_SM4574.zip › supplement/Table_S2.docx]

**Table S2. Oligonucleotides used in this study.**

| **Name** | **Sequence(5’->3’)** |
| --- | --- |
| 541 | TGGACTTGTGTTGTTATCTGGACT |
| 542 | CTTGCTGTGTTTGTGTTTGTGTTG |
| 1139 | GGAGCGGGGCCCAAGTTTAACTAAAAGTGACAAG |
| 1140 | GGAGCGCTCGAGGTGTGGATACTACAACTCCATA |
| 1141 | GGAGCGCCGCGGCAATTAATTAGTTAATGCTAGG |
| 1142 | GGAGCGGAGCTCTTTACCAACACTTTATGTGGGA |
| 1145 | CCCGAACAACAACGACAAGAACAGC |
| 1146 | CACCACCACCACCAAATCCATGATT |
| 1197 | TGATACGCCA GTTTCTTCGG |
| 1198 | TTCTGGTCGCTGTTCTTGTC |
| 1710 | GGAGCGCCGCGGGTGACAAGACGTTCACCGG |
| 1711 | GGAGCGGAGCTCCCTTCATCGATGATAAATGGCAC |
| 1781 | ATTGCCACCA ATACCTGCTC |
| 1782 | TGGCTGTCCA TGTTGTTGTT |
| 1783 | AGCAGTGCAT CAACATCCAG |
| 1784 | TCTCACACCCTTTGGACCTC |
| 1785 | CAACAACCACTGCCACAATC |
| 1786 | AGTCAGATTGCCGGAGAAGA |
| 1787 | GTATCCTGCCGCAACATCTC A |
| 1788 | GCACCACTAGGAGCACTTGT |
| 1789 | TACCAGCAGATGTCGCACTA C |
| 1790 | AATGGTCCTCGTGGTGTAATTGA |
| 1791 | TCACAACTGCTCAATACTTCATTA |
| 1792 | AAGGGTGTTGGCTATTATGC |
